# Application of halotolerant *Azotobacter chroococcum* W4ii isolated from technosoils to mitigate salt stress in wheat plant

**DOI:** 10.12688/openreseurope.15821.1

**Published:** 2023-05-12

**Authors:** Sweta Binod Kumar, Agnieszka Kalwasińska, Maria Swiontek Brzezinska, Monika Wróbel

**Affiliations:** 1 Department of Environmental Microbiology and Biotechnology, Faculty of Biological and Veterinary Sciences, Nicolaus Copernicus University in Toruń, Toruń, Kuyavian-Pomeranian Voivodeship, 87-100, Poland

**Keywords:** PGPR, Azotobacter chroococcum, Salt stress, Technosoil, Triticum aestivum L.

## Abstract

**Background:** Technosoils are soils affected by human activities. One such soil, present in Inowrocław, central Poland, is characterized by increased salinity (ECe up to 70 dS/m) and has been influenced by a soda lime repository. Microorganisms retrieved from such environments that have naturally adapted to harsh environmental conditions, especially plant growth-promoting rhizospheric (PGPR) bacteria, can help degraded soils maintain their performance. This can contribute to increased plant biomass and protection against pathogens, resulting in a reduction of mineral fertilizers and pesticides used in agriculture. The goal of this research was to obtain PGPR from degraded soil and implement them into the soil under laboratory and field conditions to improve the growth of wheat seedlings.

**Methods:** *Azotobacter chroococcum* W4ii was isolated from the rhizosphere of wheat ( *Triticum aestivum* L.) for its plant growth properties on wheat plants under salt stress.

**Results:** Wheat seeds co-inoculated with *A. chroococcum* W4ii under 200 mM salt stress showed significant improvement in various growth parameters such as plant height, shoot biomass, chlorophyll *b* content compared to un-inoculated ones. Bacterial inoculation decreased the level of malondialdehyde (MDA), whereas it elevated the antioxidative enzymatic activities of peroxidase (POD). The test isolate also enhanced the level of defense enzymes like β-1,3-glucanase, which can protect plants from the infection of pathogens. The bacterium could also successfully colonize the wheat plants.

**Conclusions:** These results indicate that *A. chroococcum* isolated from the technosoil has potential to promote wheat growth under salt stress and can be further used as bioinoculant in the salt affected agricultural fields.

## Plain language summary

Technosoil is a type of soil that is affected by human activity. In Poland, there is a technosoil that has been contaminated by a chemical storage site called soda lime. Scientists have discovered a bacteria called *Azotobacter chroococcum* W4ii in the roots of wheat plants grown in this soil. They wanted to investigate whether adding this microorganism to the soil could help wheat plants grow better. They found that adding it to the soil improved the growth of wheat plants, which grew taller, had more shoots, and contained more chlorophyll *b*. Furthermore, the bacteria helped the wheat plants fight off some pathogens. The results presented in the manuscript could lead to the development of a new preparation for sustainable agriculture.

## Introduction

Wheat ( *Triticum aestivum* L.) is the most traded and planted staple food crop globally, covering 216 million hectares with total production of 765 million tons worldwide ( FAOSTAT, 2019). However, due to climate change and various anthropogenic activities, salinity is increasing and subsequently affecting 20% of global cultivable land ( Saddiq *et al.*, 2021). The soil salinity is also distressing the wheat production leading to the yield loss up to 60% ( Dadshani *et al.*, 2019). In the European Union (especially Mediterranean countries) alone, one million hectares are affected by soil salinity ( Machado & Serralheiro, 2017).

Salt stress causes nutrient deficiency due to excessive accumulation of salt ions that inhibits the absorption of other nutrient ions by plants. Soil salinization leads to various other secondary effects like hyperosmotic stress induced water deficit and increased production of reactive oxygen species (ROS) ( Ji *et al.*, 2022).

However, application of plant growth-promoting rhizobacteria (PGPR) improves plant growth under salinity stress conditions ( Bharti *et al.*, 2016; Ji *et al.*, 2022; Singh & Jha, 2017; Upadhyay & Singh, 2015). PGPR as bio-inoculants *e.g.*, *Agrobacterium*, *Azospirillum*, *Bacillus* and *Pseudomonas* species are well established, economically feasible and environment friendly approach to reclaim salinity affected land for enhanced crop yield ( Upadhyay & Singh, 2015). Agronomic yields in response to PGPR have been increased due to the production of phytohormones (indole-3-acetic acid, gibberellic acid, zeatin, ethylene, abscisic acid), P solubilization, N fixation, siderophore and ACC deaminase enzyme production ( Abbas *et al.*, 2019). PGPR also promote plant growth by altering the selectivity of Na ^+^, K ^+^, and Ca ^2+^ and sustain a higher K ^+^/Na ^+^ ratio in plants under salt stress ( Hamdia *et al.*, 2004). Improvement in wheat cultivation in saline areas have been reported to be facilitated by the application of PGPR ( Desoky *et al.*, 2020; Nadeem *et al.*, 2013; Nawaz *et al.*, 2020).

Further, technosoils present in Inowrocław, central Poland harbor varied microbial communities. The technosoil represents a unique extreme environment that is located in the proximity of soda lime repository ponds of CIECH Soda Polska S.A ( Hulisz *et al.*, 2018). The leaching of chloride salts (mainly NaCl and CaCl _2_) into groundwater makes the soil extremely saline (ECe up to 60 dS/m) and alkaline (pH up to 8) ( Gołub & Piekutin, 2020; Hulisz & Piernik, 2013). Microorganisms naturally adapted to such extreme habitats have the potential to cope with various stresses like salinity.

Moreover, *Azotobacter* genus is a free-living, aerobic nitrogen-fixing, bacteria and the first identified species in this genus was *A. chroococcum* ( Robson *et al.*, 2015). *A. chroococcum* has been used widely as a PGPR to stimulate growth of various plant varieties under different soil types and climatic conditions ( Romero-Perdomo *et al.*, 2017). The Regulation EU 2019/1009 has recognized *Azotobacter* as one of the three bacterial genera that can be used as microbial plant biostimulant/biofertilizer ( Kerečki *et al.*, 2022). *A. chroococcum* according to different studies was found to reduce N fertilization in cotton ( Romero-Perdomo *et al.*, 2017), reduce mean germination time in sugar beet ( Kerečki *et al.*, 2022) and Hopbush Shrub ( Yousefi *et al.*, 2017), mitigate metal stress in maize ( Rizvi & Khan, 2018), increase nitrogen content of *Adathoda vasica* ( Naik *et al.*, 2007) and enhance adaptation of tomato plants under salt stress and N stress ( Van Oosten *et al.*, 2018). Other studies have suggested that it ameliorated the negative impact of NaCl in crops like maize and wheat ( Allam *et al.*, 2018; ÇAM *et al.*, 2022; Silini *et al.*, 2016).

Therefore, in the present study, an attempt was made to further explore the potential of salt-tolerant nitrogen fixing *A. chroococcum* W4ii isolated from the rhizosphere of wheat grown in the technosoils for its plant growth promoting traits in the wheat plant grown in soil under saline stress. For this, a preparation of W4ii isolate with skimmed milk was prepared and treated with the wheat plant growing under salt stress. Different biochemical and growth parameters of wheat plants were also determined to test its efficacy to confer salt stress tolerance.

## Methods

### Isolation of bacterial strain

The bacterial strain was isolated from the rhizosphere of wheat grown in the technosoils in Inowrocław, central Poland. Soil sample of 5 g was added to 45 mL of nitrogen free JMV (mannitol as the carbon source) for the enrichment of diazotrophs ( Baldani *et al.*, 2014). After seven days, 100 µl of the above prepared soil suspension was again inoculated into vials containing 5 mL of fresh semisolid JMV media and kept at 26°C. Diffuse subsurface growth pellicle appeared in vials after five days, then a loop full of culture from these vials was streaked onto nitrogen-free solid medium. Single, separated colonies growing on these plates were again streaked on the solid JMV agar plate.

Subsequently, colony PCR with primer set Pol F-Pol R ( Poly *et al.*, 2001) for *nif*H gene was performed. Positive strains for *nif*H were selected for further identification using 16S rRNA gene amplification.

### Identification of the strain

Total genomic DNA was extracted from the strain using the Gene MATRIX Bacterial & Yeast Genomic DNA Purification Kit (EURx, Gdańsk, Poland; cat. no E3580) following the instructions given by the manufacturer. The 16S rRNA gene was amplified, sequenced, and identified using the EzBioCloud database as described in our previous study ( Kalwasińska *et al.*, 2017).

### Test for plant growth promoting features and stress tolerance of *A. chroococcum* W4ii

The isolated strain was evaluated for their ability to fix N _2 _by the acetylene reduction assay (ARA) ( Beauchamp *et al.*, 1991). Briefly, each flask was closed with a Suba (Sigma-Aldrich, USA) and 10% (v/v) of the air was replaced by acetylene. The flasks were incubated for 30 min at room temperature and ethylene concentration was determined by injecting 0.1 mL of air sample in a gas chromatograph (Perkin-Elmer, Montreal, QC, Canada) equipped with a flame ionization detector (FID) and a 0.5 mm, 30 m RTQ-PLOT column (Restek, Bellefonte, PA, USA). Presence of ACC deaminase enzyme in the strain was tested by measuring the amount of α-ketobutyrate production (a cleavage product of ACC) spectrophotometrically (SpectraMax iD3 Multi-Mode Microplate Reader (Molecular Devices, San Jose, CA, USA) at 540 nm using the standard curve of α-ketobutyrate ( Ali *et al.*, 2014). Test of phosphate solubilization was performed in Pikovskaya (PVK) media agar plates supplemented with insoluble tricalcium phosphate as the phosphate source ( Sharon *et al.*, 2016). The spot cultured plate was incubated at 26°C for seven days and colonies with a clear halo were marked positive for phosphates solubilization. For IAA production test, isolated strains were propagated in DF salts minimal media supplemented with 5 mM of L-tryptophan. The supernatant of bacterial culture was then mixed with Salkowski’s reagent after 42 h of incubation. The absorbance at 535 nm was measured spectrophotometrically using a SpectraMax iD3 Multi-Mode Microplate Reader (Molecular Devices, San Jose, CA, USA) and the concentration of IAA in each culture medium was determined by comparison with a standard curve ( Patten & Glick, 2002). Test for Siderophore production was evaluated on chrome azurole S-agar (CAS-agar) plates and observed for formation of color zone around the point inoculated colony ( Schwyn & Neilands, 1987). Assay for ammonia production was tested using Ammonia Assay Kit purchased from Sigma-Aldrich (cat. no MAK310).

The tolerance of the isolate toward abiotic stressors like pH and salinity was studied. Salt tolerance (0, 1, 2 and 4 % NaCl, w/v) and pH (6-9) tolerance were tested on liquid JMV media. For both salt and pH studies, 50 μl of freshly grown culture (0.5 OD) was added to the media with different pH and salt concentration. Optical density (OD) was then determined on the seventh day with the help of densitometer DEN-18 (Biosan, Rīga, Latvia) to test the salt and pH tolerance.

### Effect of *A. chroococcum* W4ii on plant growth with and without salt treatment

Preliminary pot experiment was performed to check the efficiency of the strain on wheat plant growth in the presence and absence of salt stress. For this, seeds were treated with bacterial inoculum of 0.3 OD to avoid additional stress to the seeds. The seeds were then surface sterilized by treating with 70% ethanol followed by 2% sodium hypochlorite (NaOCl) solution for 3 min and subsequently washed using sterile water to remove all traces of sodium hypochlorite. The surface-sterilized seeds of wheat were kept in the bacterial suspension for 1 h. Surface sterilized seeds were treated with 0.03 M MgSO _4_ instead of bacterial suspension served as control. A total of 20 bacterized seeds were sown in each plastic pot (8 cm in height, 11 cm in diameter) filled with sterilized sand and vermiculite mixture grown in a controlled environment of plant growth chamber (13:11 photoperiods for 15 days at 15 ± 2°C, 1 h of dawn and dusk time and 65% light intensity *i.e.*, 1,040 klux m ^-2^). For providing salt stress and nutrient, 200 mM of NaCl was added in Hoagland medium. For comparative analysis, a set of control plants with 0 mM NaCl was taken. On the fifth day, again bacterial suspension of approximately 10 ^8^ CFU/mL was applied to the pots. Plants were watered every day after germination. The seeds were also checked in 150 mM salt concentration, where the plant growth was similar to the growth of plant under 200 mM salt concentration. Therefore, 200 mM salt concentration was selected for further experiments. Pots were arranged in completely randomized block design with three replications in each treatment.

For measuring growth (root/shoot length) and biomass (fresh/dry weight), five randomly selected plants from each replicate were used. Percentage of germination was also calculated according to the formula: *Percentage of germination* = (∑ *n* ÷ *N*) × 100 ( Fatemeh *et al.*, 2014). To estimate the chlorophyll content, fresh leaf samples of 0.1 g were homogenized with 80% acetone (10) and centrifuged at 5,000 rpm for 10 min at 4°C. The absorbance of collected supernatant were read spectrophotometrically at λ = 645 and λ = 663 nm using SpectraMax iD3 Multi-Mode Microplate Reader (Molecular Devices, San Jose, CA, USA). The same was calculated as follows ( Liang *et al.*, 2017):

Total chlorophyll (μg/mL) = 20.2 (A _645_) + 8.02 (A _663_)

Chlorophyll *a* (μg/mL) = 12.7 (A _663_) − 2.69 (A _645_)

Chlorophyll *b* (μg/mL) = 22.9 (A _645_) − 4.68 (A _663_)

### Effect of *A. chroococcum* W4ii on plant growth under salinity stress

In the pot experiment mentioned above, which used sand as the growing medium, we found no significant difference in overall plant growth, seed germination, plant weight (fresh and dry), or chlorophyll between bacteria-treated and non-treated plants in a 0 mM salt concentration. However, we observed such differences (p<0.05) under salinity conditions. Therefore, we performed further pot experiments in soil using the *A. chroococcum* W4ii formulation only in a 200 mM salt concentration.

***A. chroococcum W4ii inoculum preparation*.** The strain was grown on solid JMV medium (g L ^−1^) mannitol, 5.0; K _2_HPO _4_, 0.6; KH _2_PO _4_, 1.8; MgSO _4_.7H2O, 0.2; NaCl, 0.1; CaCl _2_.2H _2_O, 0.02; Micronutrient solution (CuSO _4_.5H _2_O, 0.04; ZnSO _4_.7H _2_O, 0.12; H _3_BO _3_, 1.40; Na _2_MoO _4_.2H _2_O, 1.0; MnSO _4_. H _2_O, 1.175. Complete volume to 1,000 mL with distilled water), 2 mL; bromothymol blue (5 g L ^−1^ in 0.2 N KOH), 2 mL; FeEDTA (16.4 g L ^−1^), 4 mL; vitamin solution (biotin, 10 mg/100 mL), 1 mL. pH was adjusted with KOH. Agar 17 g L ^−1 ^was added. Total 10 petri dishes (200 mm diameter) were used to grow the bacteria. After one week, the bacterial cell mas was collected and mixed with 10% skimmed milk. The bacterial cell mixture with skimmed milk was then lyophilized to get the powered formulation to be applied in soil. It was kept at 4°C for further use.

***A. chroococcum W4ii formulation application on wheat plants*.** The soil used for the study was autoclaved at 121°C for 1 h for three consecutive days to kill any microbial presence. Sterility of the soil was checked by standard serial dilution method. Then, 200 mM of salt was added to each pot without Hoagland medium. Seeds were surface sterilized as described above. A total of 25 seeds were sown in each pot. The prepared *A. chroococcum* W4ii formulation was dissolved in water and approximately 10 ^8^ CFU/mL was added to each pot during sowing and after five days of growth. Only water was added to pot for control treatment. Plants were sprinkled with water every day after germination. The pots were kept in the plant growth chamber with same parameter settings as aforementioned. Pots were arranged in completely randomized block design with three replications in each treatment.

After 15 days, plants were harvested for other biochemical analysis. Following uprooting, the roots were completely entangled to each other and couldn’t be separated. So, fresh and dry weight was measured for whole root system from each replicate. For measuring the biomass (fresh and dry) of shoots, 10 shoots from each replicate (pot) were measured.

### Biochemical analysis of plant

***Lipid peroxidation*.** The level of lipid peroxidation was calculated by measuring the malondialdehyde (MDA) content formed through thiobarbituric acid reaction using MDA assay kit (cat. no. MAK085) purchased from Merck (Darmstadt, Germany). For the assay, 10 mg of leaves were taken for each replicates. The MDA-TBA adduct formed was measured fluorometrically at fluorescence intensity (λ _ex_=532/λ _em_=553 nm). The calculations were done according to the kit’s specifications.

***Total soluble sugar (TSS)*.** Total soluble sugar (TSS) in control and stressed wheat leaf samples were determined according to DuBois *et al.* (1956) with some modifications. About 100 mg of fresh leaves were homogenized in 5 mL of 80% ethanol and incubated in a water bath at 80°C for 30 min. After incubation, 0.5 mL of extract was mixed with 0.5 mL of 5% phenol and 1.5 mL of 95% H _2_SO _4_ and further incubated in the dark for 15 min. Absorbance was then measured at 490 nm using the SpectraMax iD3 Multi-Mode Microplate Reader (Molecular Devices, San Jose, CA, USA).

***Proline*.** Proline content in the leaves was determined according to the standard protocol ( Bates *et al.*, 1973) with minor modifications. Fresh leaves (0.1 g) were homogenized in 1.2 mL of 3% (w/v) sulfosalicylic acid and centrifuged at 13,000 rpm for 10 min at 4°C. Resulting supernatant of 500 µL was mixed with 500 µL water and 1 mL of 2% ninhydrin. The mixture was then boiled for 30 min at 100°C. After cooling, 2 mL of toluene was added to the mixture and upper aqueous phase was used for taking absorbance at 520 nm in a spectrophotometer (SpectraMax iD3 Multi-Mode Microplate Reader (Molecular Devices, San Jose, CA, USA)). The proline content was estimated by comparing with a standard curve of L-proline as standard.

### Antioxidant assay

The peroxidase (POD) activity in the extract was determined by the method of Kar & Mishra (1976) with modifications. Plant leaves (0.5 g) were extracted in the buffer containing 5 mL of 50 mM phosphate buffer (pH 7.0) added with 1% polyvinylpyrrolidone (PVPP). The crude extract was centrifuged at 10,000 g for 15 min at 4°C, and the obtained supernatant was used for testing (POD) activity. Then, 3 mL of the assay mixture for the peroxidase activity comprised: 0.1 M phosphate buffer (pH 6), 0.027% (w/w) hydrogen peroxide, 0.5% (w/v) Pyrogallol and 0.1 mL of enzyme extract. The amount of purpurogallin formed was determined by taking the absorbance at 420 nm. In total, 100 mL of distilled water was used as blank instead of enzyme extract.

The activity was calculated according to the formula below:

Units mL ^-1^ enzyme= (A _420_ Test - A _420_ Blank)(3)(df) / (12) (0.1)

Where,

3 = Volume (in milliliters) of assay; df = Dilution factor; 12 = Extinction coefficient of 1 mg/mL of Purpurogallin at 420 nm; 0.1 = Volume (in milliliters) of crude enzyme used.

Units mg ^-1^ solid = units mL ^-1^ enzyme / mg solid mL ^-1^ enzyme.

### β-1, 3-glucanase enzyme assay

The β-1, 3-glucanase activity was determined according to Wu *et al.* (2018) by using the spectrophotometric method. The wheat roots were placed in sterile distilled water and 10-fold dilution was prepared from that. Subsequently, the roots were shaken for 20 min at 150 rpm. The supernatant of 500 µl was mixed with 500 µl of laminarin 0.5% (v/w) in 100 mM sodium acetate buffer (pH 5.5). The reaction was carried out at 50°C for 60 min and then terminated by heating for 5 min at 100°C. Next, 2 mL of dinitrosalicylate (DNS) 1% (v/w) was added to the reaction and boiled for 10 min. After the samples were cooled, the sugar concentration was measured spectrophotometrically on SpectraMax iD3 Multi-Mode Microplate Reader (Molecular Devices, San Jose, CA, USA) at 540 nm against a standard curve containing glucose as a standard (Sigma-Aldrich, USA). One unit of β-1, 3-glucanase activity (U) was defined as the amount of glucose released and expressed in μmol/mL/h.

### Root colonization efficiency of *A. chroococcum* W4ii

Root colonization of inoculated bacterium was determined after 15 days of plant growth using serial dilution plating technique on JMV-agar medium and the number of viable cells was estimated as colony forming units (CFU). For this, 100 mg of roots from both control and treated plants were washed (6–7 times) and vortexed in N-saline with glass beads.

As *A. chroococcum* W4ii has discrete morphology on the JMV nutrient plate with yellow color and black pigmentation, it was easily identifiable. But for the confirmation, random colonies with the mentioned morphology were further tested for *nif*H gene amplification.

### Statistical analysis

The experiment was conducted in completely randomized designs. The difference between each treatment and control group was analyzed by unpaired t-test or Mann-Whitney test using PAST statistical software package (version 4.03) (RRID:SCR_019129). The difference was considered significant if P ≤ 0.05.

## Results

### Identification, plant growth promotion properties and stress tolerance of the isolate W4ii

The isolate showed 99.93 16S rDNA similarity (%) with *A. chroococcum* IAM 12666 ^T^ (NCBI GenBank, accession number OL348496; Binod Kumar & Kalwasińska, 2021).

The strain was *nif*H gene positive, showed nitrogenase activity in the acetylene reduction assay, and produced ammonia and indole acetic acid (IAA). It was also salt (up to 3%) and pH (up to 9) tolerant. It was negative for ACC deaminase activity, phosphate solubilization and siderophore production.

### Growth promotion effect of *A. chroococcum* W4ii on wheat plants under saline and non-saline conditions

**Figure 1.**
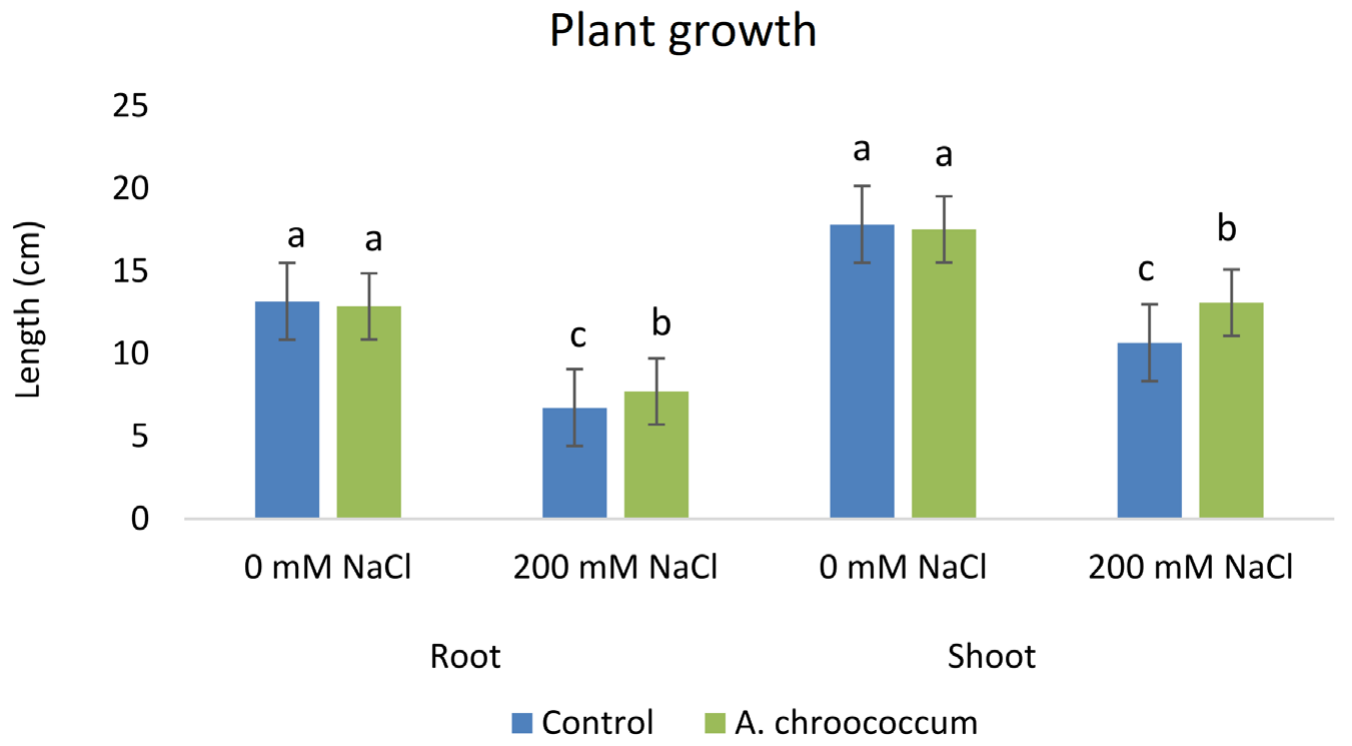
Effect of salinity on plant growth in the sand. Each data represent the mean ± SD of triplicate sets of five measurements (n = 15). Different letters on the bar in each column represent the significant difference.

**Figure 2.**
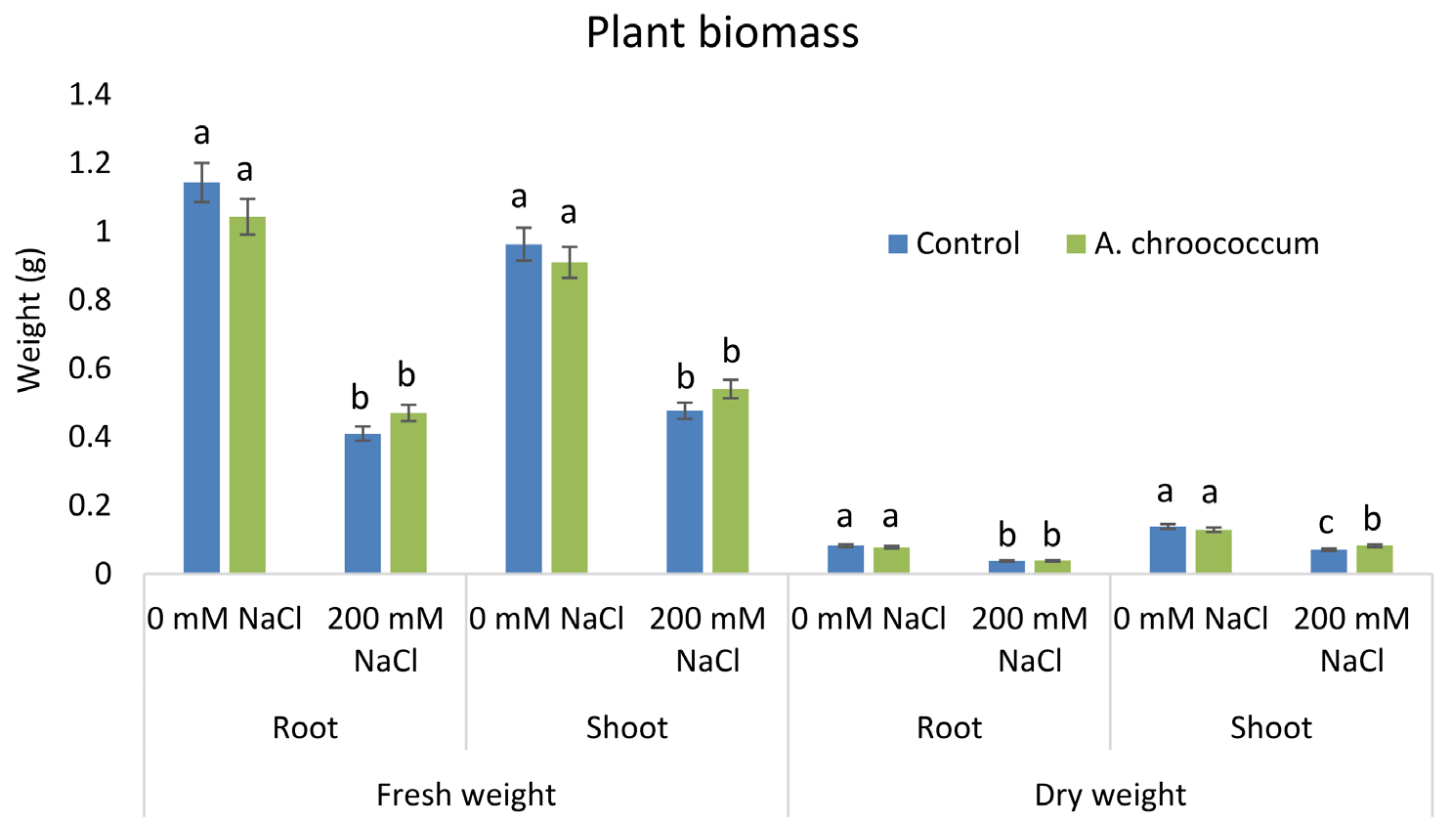
Effect of salinity on plant biomass in the sand. Each data represent the mean ± SD of five plants in triplicate sets (n = 3). Different letters on the bar in each column represent the significant difference.

In the preliminary study of the pot experiment with sand, no significant difference was found in the growth between control and bacterial inoculated wheat plants grown without any salt stress ( Figure 1 and Figure 2) ( Kalwasińska, 2023).

**Figure 3.**
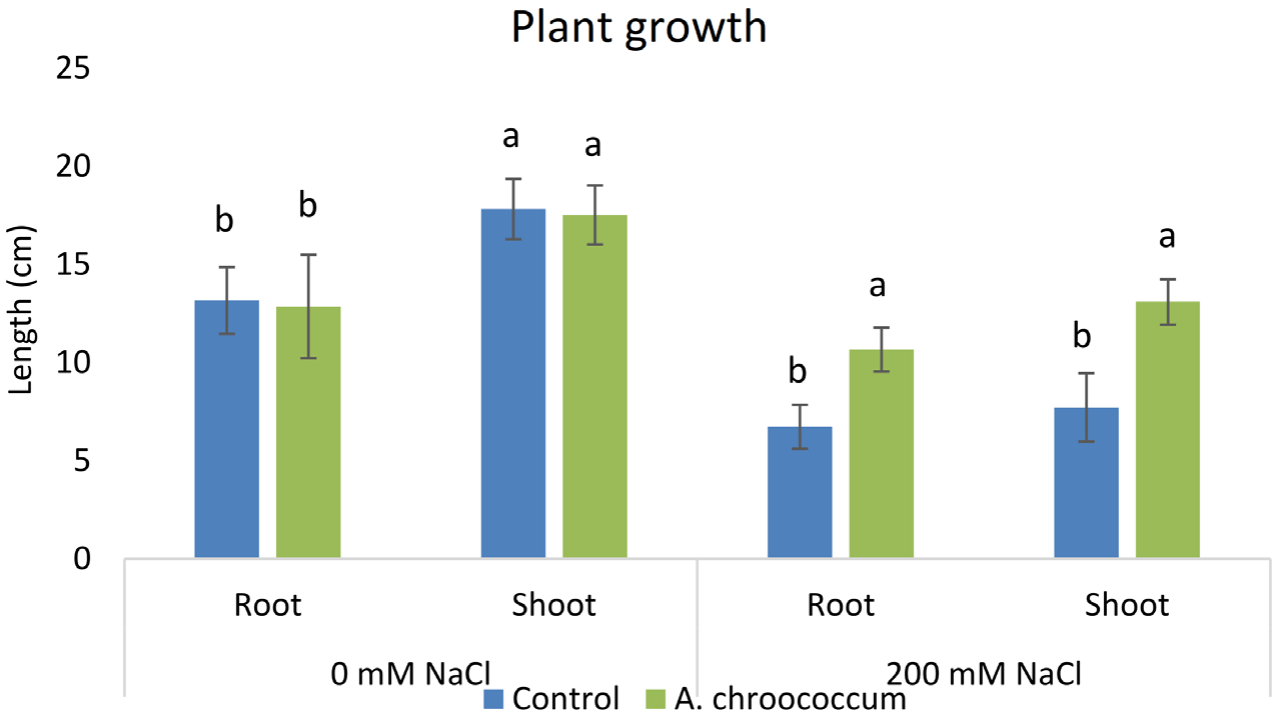
Effect of inoculation of *A. chroococcum* W4ii on plant growth in the sand under 0 mM NaCl and 200 mM NaCl. Each data represent the mean ± SD (n = 15). Different letters on the bar in each column represent the significant difference (P<0.05).

In 200 mM salt stress in soil, a significant increase at P < 0.05 was there in both root and shoot length in the bacteria treated plant in comparison to the control ( Figure 3).

**Figure 4.**
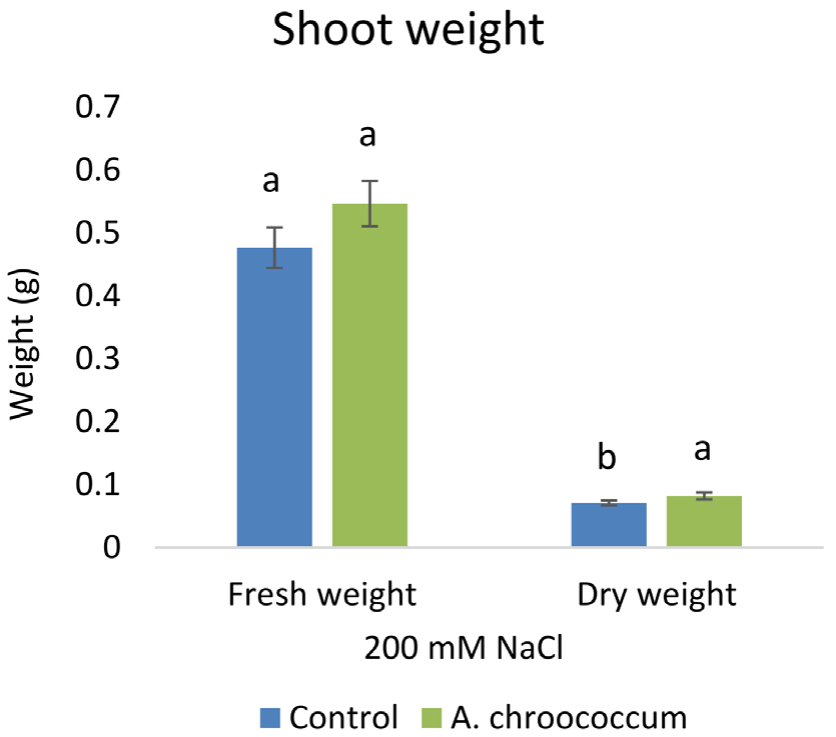
Effect of inoculation of *A. chroococcum* W4ii on plant shoot weight in the sand under 200 mM NaCl. Each data represent the mean ± SD of 5 plants in triplicate sets (n = 3). Different letters on the bar in each column represent the significant difference (P<0.05).

**Figure 5.**
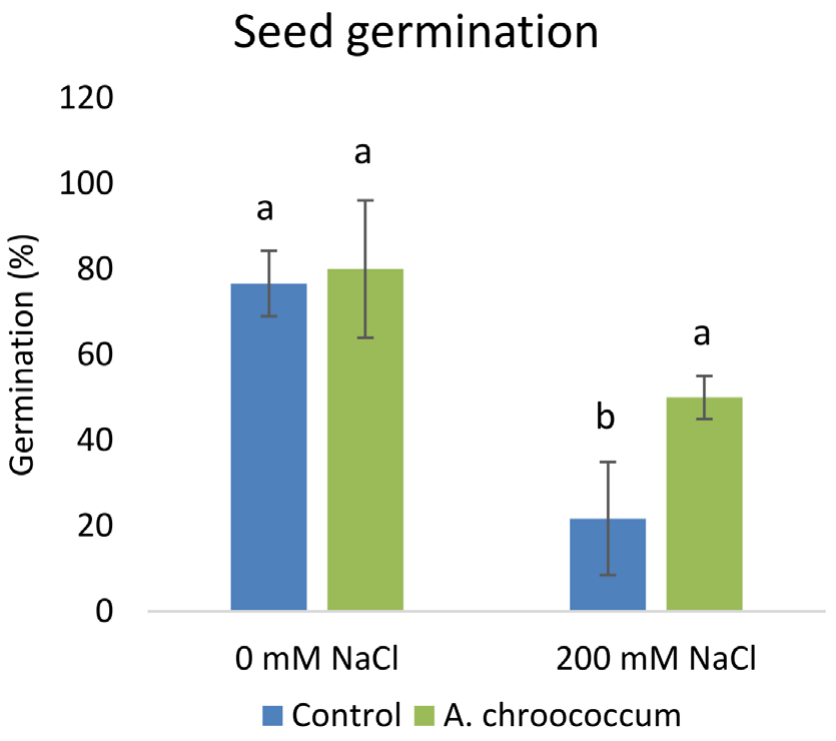
Seed germination in the sand. Each data represent the mean ± SD (n = 3). Different letters on the bar in each column represent the significant difference (P<0.05).

**Figure 6.**
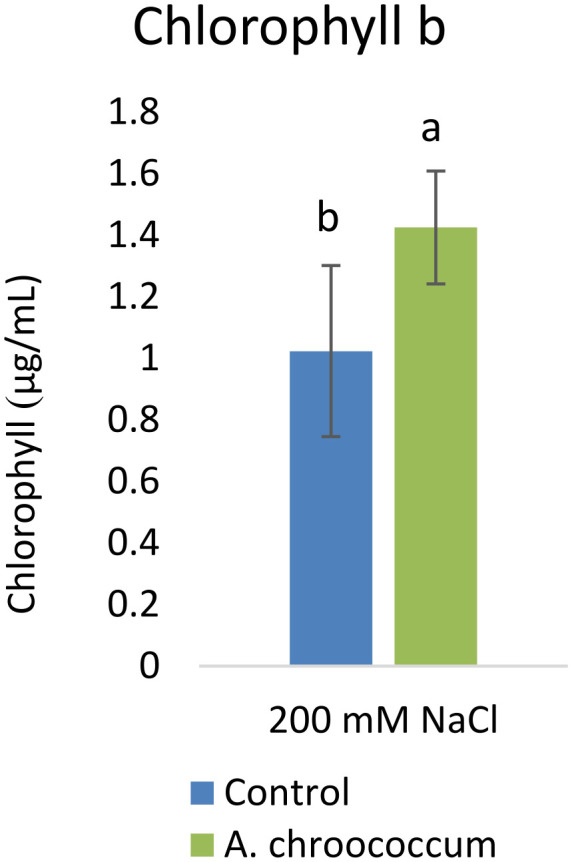
Effect of inoculation of *A. chroococcum* W4ii on plant chlorophyll *b* under 200 mM NaCl in the sand. Each data represent the mean ± SD (n = 6). Different letters on the bar in each column represent the significant difference (P<0.05).

Also, there was a significant increase (P < 0.05) in the shoot biomass (dry) treated with bacteria under salt stress ( Figure 4). However, there was no improvement in the root biomass under both 0 mM and 200 mM salt treatment when inoculated with bacteria. The percentage of germination was also significantly higher (P < 0.05) in the pots treated with the isolate compared to the non-treated ones provided with 200 mM salt concentration ( Figure 5). Then again, in the soil without salt; there was no difference in the percentage of germination between control and bacteria treated pots. Also, there was a significant (P < 0.05) increase in chlorophyll *b* content in the bacteria treated plants compared to control plants ( Figure 6).

### Efficiency of *A. chroococcum* W4ii formulation on wheat plant grown in soil under salinity stress

**Figure 7.**
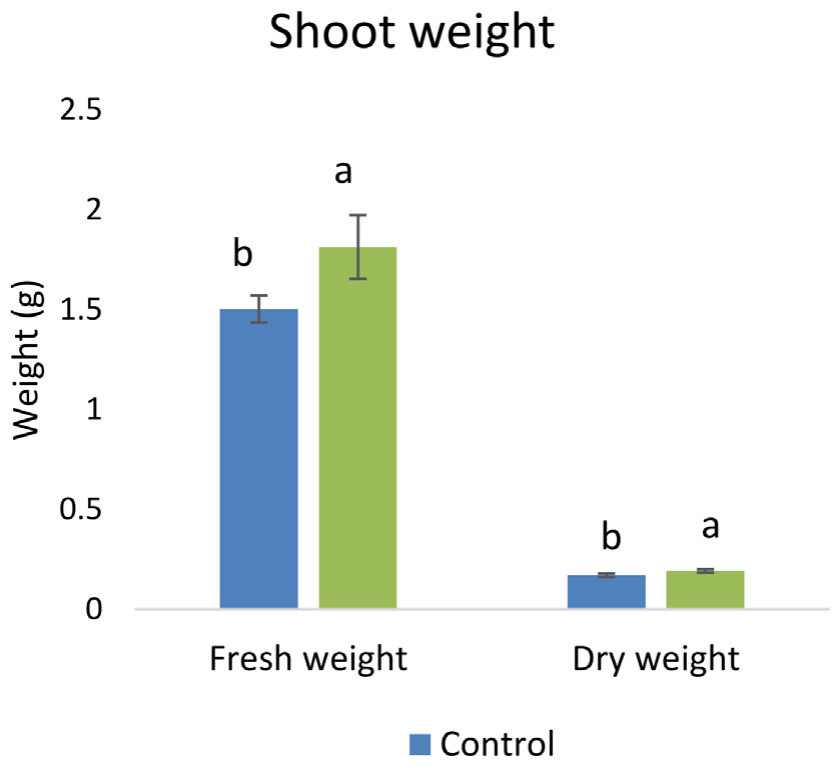
Effect of inoculation of *A. chroococcum* W4ii on plant shoot weight under 200 mM NaCl in soil. Each data represent the mean ± SD of 10 plants in triplicate sets (n = 3). Different letters on the bar in each column represent the significant difference.

The pot experiments in the sterile sand clearly showed that there was no effect of *A. chroococcum* W4ii on wheat plant growth without salinity stress. It could only protect and promote the growth of plants under saline conditions. Therefore, further experiments were done in the soil environment provided with the salinity stress of 200 mM NaCl. In the given stress, the isolate significantly (P<0.05) increased the shoot biomass (both fresh and dry) by one fold approximately ( Figure 7).

**Figure 8.**
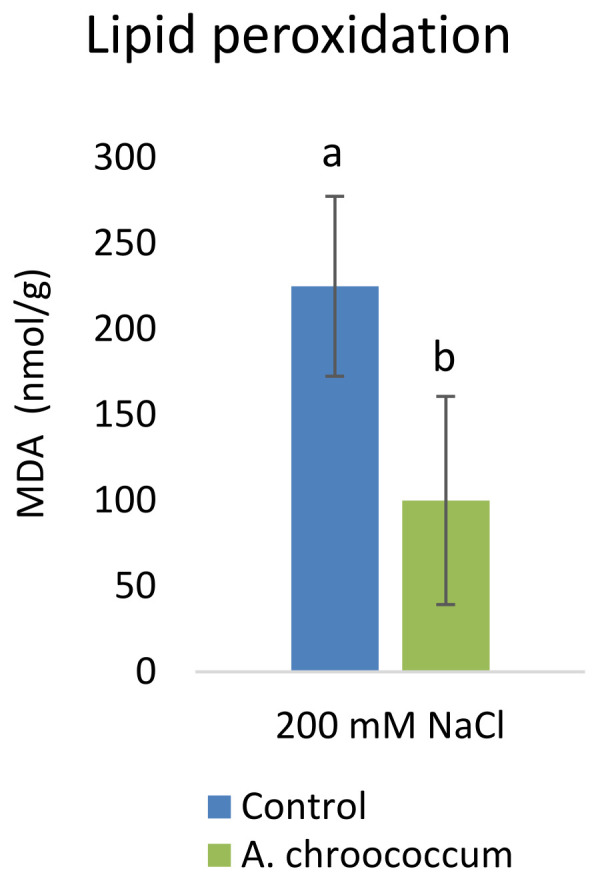
Effect of inoculation of *A. chroococcum* W4ii on plant MDA content under 200 mM NaCl in soil. Each data represent the median ± IQR (n = 9). Different letters on the bar in each column represent the significant difference (P<0.05).

Inoculation with *A. chroococcum* W4ii significantly reduced the MDA content by 55.5% (P<0.001) under the salt stress condition ( Figure 8).

**Figure 9.**
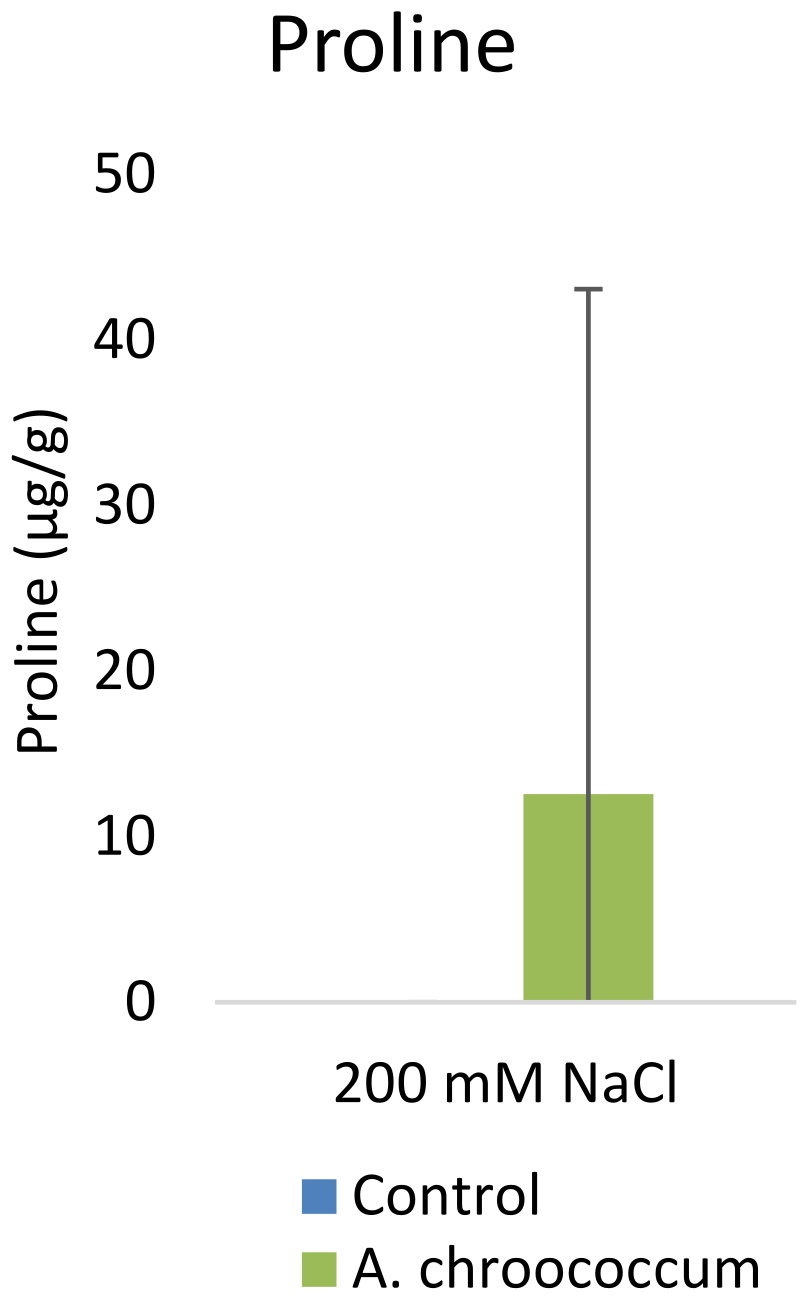
Effect of inoculation of *A. chroococcum* on plant proline content under 200 mM NaCl in soil. Each data represent the median ± IQR of triplicate sets (n = 3).

**Figure 10.**
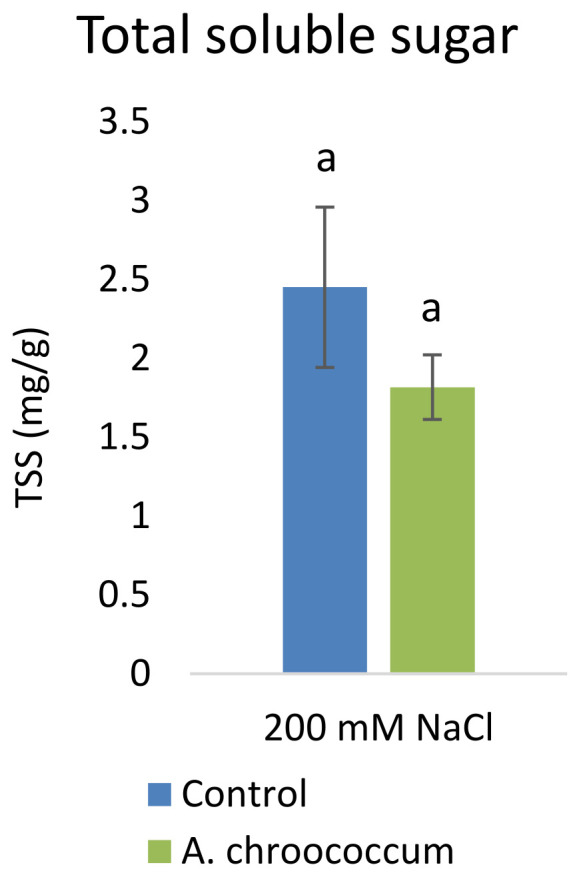
Effect of inoculation of *A. chroococcum* W4ii on plant total soluble sugar (TSS) under 200 mM NaCl in soil. Each data represent the mean ± SD of triplicate sets (n = 3). The same letters on the bar in each column represent no significant difference.

However, there was no significant difference in the proline and TSS content after bacterial inoculation compared to the control ( Figure 9 and Figure 10).

**Figure 11.**
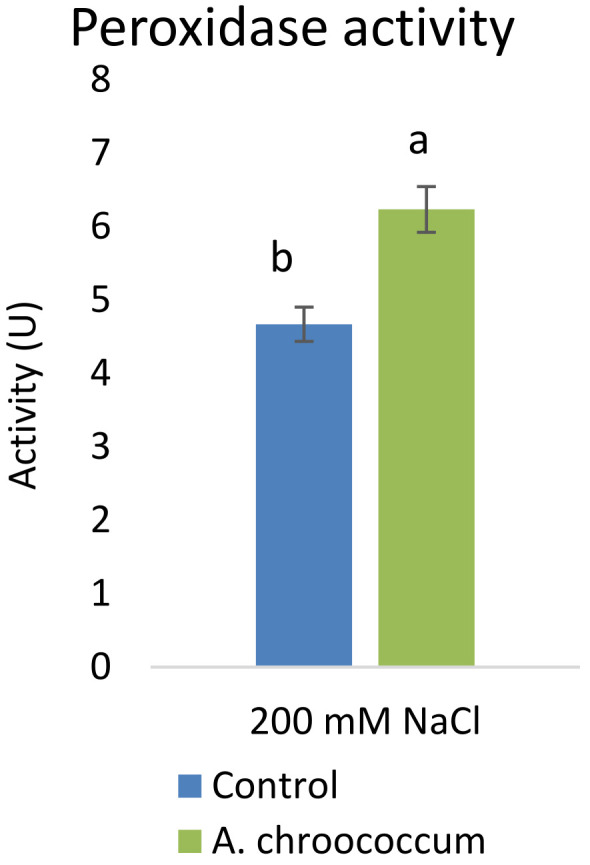
Effect of inoculation of *A. chroococcum* W4ii on plant’s peroxidase activity under 200 mM NaCl in soil. Each data represent the mean ± SD (n = 9). Different letters on the bar in each column represent the significant difference (P<0.05).

**Figure 12.**
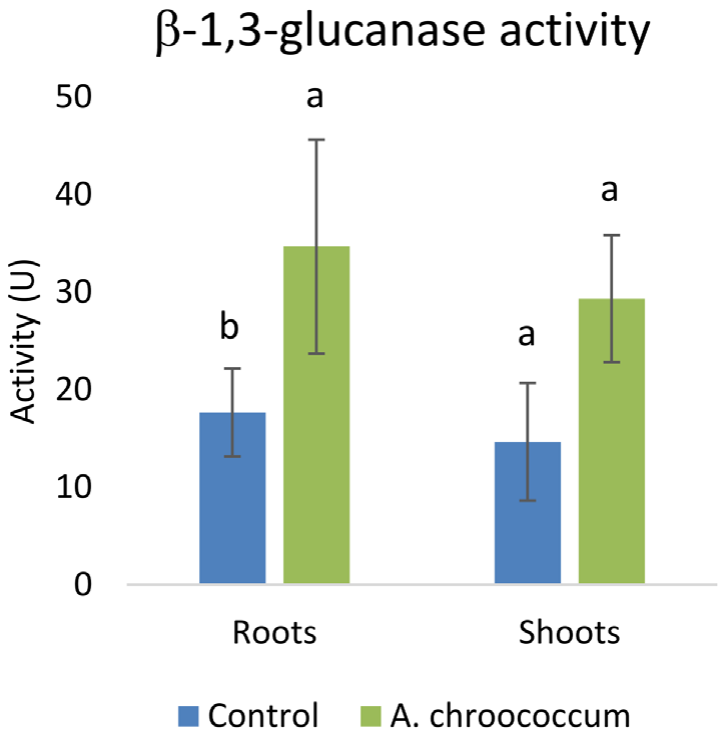
Effect of inoculation of *A. chroococcum* W4ii on root and shoot of plant’s β-1,3-glucanase activity under 200 mM NaCl in soil. Each data represent the mean ± SD (n = 3). Different letters on the bar in each column represent the significant difference (P<0.05).

Again, inoculation with the isolate significantly (P<0.001) increased the antioxidative activities *i.e.*, peroxidase activity by 33.69% to alleviate the salinity induced free radical damages ( Figure 11). In addition to that, there was a two-fold (P<0.05) increase in the defense enzymes *i.e.*, glucanase in the roots of wheat plants treated with the W4ii compared to the control ones ( Figure 12).

### Root colonization

After 15 days of plant growth, the associated *A. chroococcum* W4ii was found in a range of 10 ^5^ CFU/g of the root. No bacterial colonies were recovered from uninoculated control plants. The *nif*H gene amplification also confirmed that the bacterium was N _2_-fixer and indicated that the bacterium had successfully colonized the plant’s root system.

## Discussion

In the present work, PGPR activity of *A. chroococcum* W4ii and its effect on wheat plant under salinity stress was studied in detail. The bacterium was isolated from the rhizosphere of wheat plant grown in technogenic soil affected by soda industry and thus had inherent resistance to harsh environments. The Polish winter wheat variety (Ostroga) was chosen for the experiments because it has good grain quality, high cropping power and frost resistance ( Danko, 2010). Since, the wheat variety is frost resistant, it could be grown very well in the reclaimed or salt affected soils with the PGPR that can help it to better adapt in saline fields as well.

Our study showed that the isolated PGPR did not have any significant effect on the wheat variety in non-saline conditions. Another reason may be the Na ^+^-dependent growth of *A. chroococcum*, which has been described for several strains including ATCC 7493 and the type strain ATCC 9043 ( Robson *et al.*, 2015). Similarly, a detailed study by Page (1986) concluded that it is important to determine the geographic distribution of Na ^+^-dependent strains of *A. chroococcum* if they are associated to particular soil types and aware of the Na ^+^ requirements of the strain if they are unsuited to soil conditions ( *i.e.*, the soil has low exchangeable Na ^+^). Another investigation also revealed that *A. chroococcum* AZ6 did not show any improvement in plant length in non-saline conditions for Waha ( *Triticum durum* Waha *lcv*) wheat variety ( Silini *et al.*, 2016). Thus, both Na ^+^-dependency of strains and wheat variety could affect growth promotion in non-saline soil.

However, the wheat variety was not salt resistant as the results from pot trials with sand indicated that the growth of wheat was seriously affected by salinity stress. All the growth parameters of plants *i.e.*, shoot length, root length, shoot (fresh and dry) and root (fresh and dry) weight significantly decreased under 200 mM salt stress. But after treatment with salt-tolerant *A. chroococcum* W4ii, the growth parameters of wheat were significantly improved under salt stress. It showed consistent results in the improvement of plant length (both root and shoot) and shoot biomass (both fresh and dry) in both sand and soil.

Notably, the fresh and dry weight of the shoot increased after bacterial treatment and there was no effect on the root biomass. In general, the root/shoot ratio is proportional to nutrient supply/fertilization, with a greater ratio at low nutrient supply according to the concept of functional equilibrium. This theory was further approved by Nobel *et al.* (1989) with their study on *Agave lechuguilla*, where N application ha ^-l^ increased the shoot dry weight with no effect on root dry weight and reduced the root/shoot ratio. Similarly, Bonifas *et al.* (2005), conferred that the root: shoot ratio decreased for both corn and velvetleaf as a result of increased N supply. Yet again, *A. chroococcum* is known to reduce N-fertilization in many plant species *e.g.*, in *Adathoda vasica* Nees. ( Naik *et al.*, 2007), cotton ( Romero-Perdomo *et al.*, 2017), maize ( Meshram & Shende, 1982) and tomato ( Van Oosten *et al.*, 2018) Therefore, it can be interpreted that the isolate W4ii also has the capability of providing nitrogen to the plant and subsequently reducing N-fertilization.

The bacterium not only improved the growth parameters but also helped in the germination of seeds. The percentage of germination was significantly higher (by 130%) in the pots treated with bacteria compared to the control ones. The isolate induced greater germination possibly due to the ability of PGPR to synthesize hormones such as indole acetic acid and gibberellic acid that regulate cell division favoring seed germination ( Pérez-García *et al.*, 2023). W4ii also showed the production of IAA that helped in the germination process of seeds. Previous studies conducted by Yousefi *et al.*, 2017 also revealed that under saline conditions (at 20 and 50 dS/m), *A. chroococcum* treatment led to improved germination rate and percentage in hopbush seeds compared to the control. Yet again, *A. chroococcum* loaded nanofiber improved the germination percentage of green gram seeds in the separate study conducted by Kumuthan *et al.*, 2023.

Of note, only chlorophyll *b* content was significantly increased (approximately 40%) after bacterial treatment in the salt treated plants. But there was no significant effect on total chlorophyll content. This finding was very new up to our understanding. However, a detailed study by Biswal *et al.*, 2012 on tobacco plant showed that the increased chlorophyll *b* synthesis led to an increase in the capture of light and enhanced (40–80%) electron transport rates of photosystems I and II at both limiting and saturating light intensities. An increase in the light saturated photosynthetic carbon assimilation, starch content, and dry matter accumulation was also reported. Increased level of chlorophyll *b* additionally showed delayed senescence, leading to a longer period of photosynthesis and biomass production. Therefore, application of isolated *A. chroococcum* W4ii strain as a biostimulant may help plants to have longer photosynthetic periods and improved productivity. At this point, more studies are needed.

Salt stress increases the generation of ROS in plants, which enhances the membrane lipid peroxidation and increases the MDA content. When plants are subjected to salinity, MDA is deposited in tissues to indicate membrane destruction ( Mousavi *et al.*, 2022; Parida & Das, 2005). Therefore, leaf MDA content is usually used to estimate plant tolerance to salinity ( Katsuhara *et al.*, 2005; Luna *et al.*, 2000). The decrease in MDA content in W4ii inoculated plants indicated that bacterial inoculation protected the plants from the imposed salt stress.

Proline accumulation in response to salt stress protects the cell membrane by adjusting intracellular osmotic pressure ( Claussen, 2005). Accumulation of compatible osmolytes such as soluble sugars also helps plants to overcome abiotic stresses by maintaining osmotic turgor ( Grover *et al.*, 2014). However, our results showed no significant change in proline and sugar content after inoculation under salt stress. In another way, it can be interpreted that the plant could establish other salt tolerance mechanisms such as the production of antioxidant enzymes, stress hormones, and the overexpression of genes involved in salt stress tolerance *e.g.*, up-regulation of the aquaporin gene family ( Gond *et al.*, 2015). This can be explained by our results, which showed increased production of antioxidant enzyme (POD) utilizing an alternative mechanism to resist salt stress. Further, in a similar study by Abdel Latef *et al.*, (2021) *A. chroococcum* strain mitigated salt stress in canola plants grown in saline soil but the inoculation did not show any difference in proline and soluble sugar contents; however, there was an increase in POD activity by 121.7%.

PGPR are also known to mediate induced systemic resistance, which is associated with the induction of various defense enzymes like β-1, 3-glucanase, phenylalanine ammonia-lyase (PAL), polyphenol oxidase (PPO), and peroxidase (PO). Accumulation of pathogenesis-related (PR) proteins viz. β-1, 3-glucanase is known to be associated with systemic acquired resistance (SAR) in plants ( Meena *et al.*, 2000). Various studies have shown that PR-proteins are induced in plants upon treatment with PGPR ( Adhipathi *et al.*, 2014; Meena *et al.*, 2000; Singh & Jha, 2017). The production of various defense enzymes in the presence of W4ii might illustrate its role in the generation of resistance to pathogen infection. The increased level of β-1, 3-glucanase in the plant roots might play a key role in pathogen suppression in bacterium-primed plants. Again, β-1, 3-glucanase have the potential to hydrolyze β-1, 3-glucanase, which is a major component of the fungal cell wall. Moreover, glucanase release elicitors from the walls of fungi, which, in turn, stimulate various defense responses in plants ( Meena *et al.*, 2000). Therefore, plants can be challenged with fungal pathogen to further evaluate the improvement of plant’s ISR when treated with *A. chroococcum* W4ii. Our results also corroborate with the research work where *A. chroococcum* treated plants showed higher peroxidase and β-1, 3-glucanase enzyme activities in the cucumber plants ( El-Borollosy & Oraby, 2012).

Finally, the isolated strain W4ii showed efficient colonization to the plant root. It indicates that the ability of colonization of the isolate can provide the concentrated benefits to plants as it creates a close relationship with the plant host than rhizosphere and modulates the defense response to cope with hostile conditions. For more prolonged activity the bacterium needs to have better survival, which is achieved by effective colonization that provides protective environment ( Hardoim *et al.*, 2008).

Apart from raising nutrient availability to plants and various PGPR properties, *A. chroococcum* W4ii produces cell-associated black pigment known as catechol melanin, which is thought to be a protective mechanism for aeroadaptation in Na ^+^ dependent strains/minimizing oxidative stress ( Shivprasad & Page, 1989). Many research studies have concluded that the bacterium provides salinity tolerance to various plant species especially in wheat ( Alkhalifah *et al.*, 2018; Allam *et al.*, 2018; Chaudhary *et al.*, 2013; Silini *et al.*, 2016), which is in accordance with our present study. In addition to that, the strain has also been used in consortium with other PGPR for further improvement in plant growth and stress resistance ( Alkhalifah *et al.*, 2018; Kumar Bhuyan *et al.*, 2015; Yousefi *et al.*, 2017).

## Conclusions

Technosoils are valuable sources of salt tolerant PGPR. In the present study, *A. chroococcum* W4ii strain isolated from the technosoils significantly improved the biochemical parameters of winter wheat plants and thus allowed them to cope with imposed salinity stress. The bacterium can very well be used for growing crops in the reclaimed land with salinity problems. Use of this PGPR may be ideal for reducing chemical fertilizer in the agricultural fields to reduce its impact on environmental health. Source of the isolation also makes this strain more robust as it can survive extreme environments and its reproducibility of results to mitigate salt stress makes it a perfect tool for increasing wheat yield under salinity conditions. Further research at the molecular level will allow us to better understand the plant-microbe interaction for example, the increase in chlorophyll *b* content after *A. chroococcum* W4ii treatment.

## Ethics and consent

Ethical approval and consent were not required.

## Data Availability

NCBI GenBank: *Azotobacter chroococcum* strain W4ii 16S ribosomal RNA gene, partial sequence. Accession number OL348496, https://identifiers.org/ncbi/insdc:OL348496 ( Binod Kumar & Kalwasińska, 2021). RepOD: Application of halotolerant Azotobacter chroococcum W4ii isolated from technosoils to mitigate salt stress in wheat plant. https://doi.org/10.18150/10SRTX ( Kalwasińska, 2023). Data are available under the terms of the Creative Commons Zero "No rights reserved" data waiver (CC0 1.0 Public domain dedication).
