# Supplementary material for: Using halotolerant Azotobacter chroococcum W4ii from technosoils to mitigate wheat salt stress
Source: Open Res Eur. 2024 Sep 9;3:76. Originally published 2023 May 12. [Version 4] doi: 10.12688/openreseurope.15821.4 (PMC11325138; doi:10.12688/openreseurope.15821.4)
Supplement: Peer review history

# Reviewer report: Reviewer response for version 4

**DOI:** 10.21956/openreseurope.20020.r44062

**Published:** 2024-09-10

**Authors:** Hemant Dasila

**Affiliations:** 1 Eternal University, Baru Sahib, Himachal Pradesh, India; 2 G.B. Pant University of Agriculture and Technology, Pantnagar, India

Manuscript is refined in a good manner after revision and fit for indexing from my end.

Is the study design appropriate and does the work have academic merit?

Yes

Is the work clearly and accurately presented and does it cite the current literature?

Yes

If applicable, is the statistical analysis and its interpretation appropriate?

No

Are all the source data underlying the results available to ensure full reproducibility?

Yes

Are the conclusions drawn adequately supported by the results?

Yes

Are sufficient details of methods and analysis provided to allow replication by others?

Yes

Reviewer Expertise:

Agriculture Microbiology, Applied Microbiology, Biocontrol, Bioremediation, Microbial Biotechnology and Abiotic stress.

I confirm that I have read this submission and believe that I have an appropriate level of expertise to confirm that it is of an acceptable scientific standard.

# Reviewer report: Reviewer response for version 3

**DOI:** 10.21956/openreseurope.19283.r42677

**Published:** 2024-08-14

**Authors:** Hemant Dasila

**Affiliations:** 1 Eternal University, Baru Sahib, Himachal Pradesh, India; 2 G.B. Pant University of Agriculture and Technology, Pantnagar, India

Authors have tried to address the issue of high salinity in the region which is affecting plant growth and promotion. Overall manuscript is fit for indexing with some small queries from my end and some minor changes as follows;

 1) Abstract is kind of general it should be precise according to the results and these results should be summarized in abstract.

 2) Authors have used the term hypothesis which may be null or alternative hypothesis. Did Authors have applied any statistical analysis to test the hypothesis.

Is the study design appropriate and does the work have academic merit?

Yes

Is the work clearly and accurately presented and does it cite the current literature?

Yes

If applicable, is the statistical analysis and its interpretation appropriate?

No

Are all the source data underlying the results available to ensure full reproducibility?

Yes

Are the conclusions drawn adequately supported by the results?

Yes

Are sufficient details of methods and analysis provided to allow replication by others?

Yes

Reviewer Expertise:

Agriculture Microbiology, Applied Microbiology, Biocontrol, Bioremediation, Microbial Biotechnology and Abiotic stress.

I confirm that I have read this submission and believe that I have an appropriate level of expertise to confirm that it is of an acceptable scientific standard.

# Response

**Published:** 2024-09-03

**Authors:** Agnieszka Kalwasińska

**Affiliations:** Department of Environmental Microbiology and Biote, Nicolaus Copernicus University in Toruń, Torun, Poland

Dear Professor Hemant Dasila,   Thank you for taking the time to review our work and for providing valuable feedback. Your comments have been helpful in improving our manuscript. Please find our responses to your comments below:

 1) Abstract is kind of general it should be precise according to the results and these results should be summarized in abstract.

 Answer: The results have been summarized and highlighted in the improved abstract part as per your suggestion.

 2) Authors have used the term hypothesis which may be null or alternative hypothesis. Did Authors have applied any statistical analysis to test the hypothesis.

 Answer: Yes, the statistical analysis was performed for the experiments conducted in the present work and described under the title “ **Statistical analysis**” in Methods section. The difference between each treatment and control group was analyzed by unpaired t-test or Mann-Whitney test using PAST statistical software package (version 4.03) (RRID:SCR_019129). The difference was considered significant if P ≤ 0.05. Best regards, Agnieszka Kalwasińska

# Reviewer report: Reviewer response for version 2

**DOI:** 10.21956/openreseurope.17765.r37347

**Published:** 2024-05-20

**Authors:** Kailash Chand Kumawat

**Affiliations:** 1 Department of Industrial Microbiology, Jacob Institute of Biotechnology and Bioengineering, Sam Higginbottom University of Agriculture Technology and Sciences, Allahabad, Uttar Pradesh, India

**Title:** Title should be reframed and shortened according to study.

**Abstract:** Abstract needs to be reframed. Also, add field’s trial experiment with multifarious PGP traits (at least two years trials based study) and compare under normal soil a minimum of two years field study will be required and then conclude your study because field environmental condition are totally different from pot experiment.

**Introduction:** Introduction part is very simple and descriptive, so that improve your introduction part with some mechanistic part and justify your study properly with outcomes.   

**Material and methods:** Material and methods are not up to date, please add your two years field study and compare in the normal soil conditions with various plant growth attributes, symbiotic efficiency.

**Results:** Results are not up to date.  Results should be reframe and try to short with important results in the text and remaining mention in the tables and figures. Check all the tables and figures in the text carefully.

**Discussion:** Discussion part is very poor, improve discussion part and support your study with mechanistic part. Add only recent study in the discussion part to support study with mechanistic part.

**Conclusion:** Conclusion should be modified after incorporation of all the suggestions, field study results for the farmer communities.

**References:** References should be reframed according to journal guidelines and check carefully all references in the text as well as references list.

**Overall recommendations:** According to my opinion this study not completed without two years field study and compared with normal and saline soil conditions under field condition because field environmental condition are totally different from pot study.

Is the study design appropriate and does the work have academic merit?

No

Is the work clearly and accurately presented and does it cite the current literature?

No

If applicable, is the statistical analysis and its interpretation appropriate?

Partly

Are all the source data underlying the results available to ensure full reproducibility?

Partly

Are the conclusions drawn adequately supported by the results?

Partly

Are sufficient details of methods and analysis provided to allow replication by others?

No

Reviewer Expertise:

Plant Microbial Interactions

I confirm that I have read this submission and believe that I have an appropriate level of expertise to state that I do not consider it to be of an acceptable scientific standard, for reasons outlined above.

# Response

**Published:** 2024-05-22

**Authors:** Agnieszka Kalwasińska

**Affiliations:** Department of Environmental Microbiology and Biote, Nicolaus Copernicus University in Toruń, Torun, Poland

Dear Professor Kailash Chand Kumawat,

 Thank you very much for your valuable comments on our manuscript. Here, we present our responses to your comments.

 Title: Title should be reframed and shortened according to study. Using halotolerant *Azotobacter chroococcum* W4ii from technosoils to mitigate wheat salt stress

 Ans: The title has been reframed and shortened accordingly.

 Abstract: Abstract needs to be reframed. Also, add field’s trial experiment with multifarious PGP traits (at least two years trials based study) and compare under normal soil a minimum of two years field study will be required and then conclude your study because field environmental condition are totally different from pot experiment.

 Ans: Thank you for the suggestion. However, the project related to this study has concluded, so the suggested study cannot be continued. More importantly, this study was part of a larger research project where a range of PGPR were isolated from the technosoil. Other novel strains were also selected as potential bio-stimulants and subsequently chosen for trial-based studies under field conditions. *Azotobacter chroococcum* is an established PGPR; therefore, we have only reported its presence in the technosoil as a potential PGPR. It can be further studied for a field trial.

 Introduction: Introduction part is very simple and descriptive, so that improve your introduction part with some mechanistic part and justify your study properly with outcomes.  

 Ans: Thank you for the suggestion. However, our present study was only a part of the project and indeed quite simple. We did not use any mechanistic approach, and the introduction section aptly describes the idea behind this study..

 Material and methods: Material and methods are not up to date, please add your two years field study and compare in the normal soil conditions with various plant growth attributes, symbiotic efficiency.

 Ans: As stated above.

 Results: Results are not up to date.  Results should be reframe and try to short with important results in the text and remaining mention in the tables and figures. Check all the tables and figures in the text carefully.

 Ans: All the tables and figures in the text have been carefully checked as per your suggestion. Only the important results are mentioned in the text.

 Discussion: Discussion part is very poor, improve discussion part and support your study with mechanistic part. Add only recent study in the discussion part to support study with mechanistic part.

 Ans: As stated above, the present study cannot be continued, and no mechanistic research has been conducted in this study.

 Conclusion: Conclusion should be modified after incorporation of all the suggestions, field study results for the farmer communities.

 Ans: As stated above, the present study cannot be continued further as it was only a part of a project that is already completed.

 References: References should be reframed according to journal guidelines and check carefully all references in the text as well as references list.

 Ans: The references have been checked as per your suggestion and are in accordance with the journal guidelines.

 Overall recommendations: According to my opinion this study not completed without two years field study and compared with normal and saline soil conditions under field condition because field environmental condition are totally different from pot study.

 Ans: We completely agree with your opinion regarding our present manuscript. However, A. chroococcum has already been extensively studied as a PGPR. Therefore, we limited our study to pot experiments. In our conclusion, we mentioned that further studies could be conducted in real-world agricultural settings with varying levels of salinity and other scopes. Separately, we have conducted field studies with other novel strains isolated from the same technosoils. In the future, we can propose another project to continue our studies based on your suggestions. We thank you for your valuable comments.

# Reviewer report: Reviewer response for version 2

**DOI:** 10.21956/openreseurope.17765.r34425

**Published:** 2023-08-15

**Authors:** Megha Kaviraj

**Affiliations:** 1 University of Birmingham, Birmingham, England, UK

Yes, the authors' significant modifications and revisions can be now approved for scientific standard publication.

Is the study design appropriate and does the work have academic merit?

Yes

Is the work clearly and accurately presented and does it cite the current literature?

Yes

If applicable, is the statistical analysis and its interpretation appropriate?

Yes

Are all the source data underlying the results available to ensure full reproducibility?

Partly

Are the conclusions drawn adequately supported by the results?

Partly

Are sufficient details of methods and analysis provided to allow replication by others?

Yes

Reviewer Expertise:

Biological nitrogen fixation, plant-microbe interaction, soil microbiology, Biofertilizer, Nitrogen cycle, DNRA, Molecular Biology, Metagenomics, Bioinformatics

I confirm that I have read this submission and believe that I have an appropriate level of expertise to confirm that it is of an acceptable scientific standard.

# Reviewer report: Reviewer response for version 1

**DOI:** 10.21956/openreseurope.17091.r33560

**Published:** 2023-07-24

**Authors:** Megha Kaviraj

**Affiliations:** 1 University of Birmingham, Birmingham, England, UK

1. The article is quite an interesting and details study. However, the abstract should be more focused on a line of research gap, hypothesis and a few best quantitative percentages of improvement data.
2. The authors should specify in the methods section at what point or how many days after wheat growth a soil sample was taken. Additionally, the fertilizer regime for that scenario should be included.
3. The data that was not presented in the bar graph can be incorporated in tabular form for better understanding.
4. The organism’s name should be italics, please rectify it throughout the manuscript including graphs.
5.  “The bacterial cell mass was collected and mixed with 10% skimmed milk. The bacterial cell mixture with skimmed milk was then lyophilized to get the powered formulation to be applied in soil”. What is the reason behind using carrier-based solid formulation instead of liquid formulation? How economical and viable to make it solid with skimmed milk?
6. The last line of future study in the conclusion section is very generalized please make it robust.
7. Please check spelling and English corrections for better readability.

Is the study design appropriate and does the work have academic merit?

Yes

Is the work clearly and accurately presented and does it cite the current literature?

Yes

If applicable, is the statistical analysis and its interpretation appropriate?

Yes

Are all the source data underlying the results available to ensure full reproducibility?

Partly

Are the conclusions drawn adequately supported by the results?

Partly

Are sufficient details of methods and analysis provided to allow replication by others?

Yes

Reviewer Expertise:

Biological nitrogen fixation, plant-microbe interaction, soil microbiology, Biofertilizer, Nitrogen cycle, DNRA, Molecular Biology, Metagenomics, Bioinformatics

I confirm that I have read this submission and believe that I have an appropriate level of expertise to confirm that it is of an acceptable scientific standard, however I have significant reservations, as outlined above.

# Response

**Published:** 2023-07-28

**Authors:** Agnieszka Kalwasińska

**Affiliations:** Department of Environmental Microbiology and Biote, Nicolaus Copernicus University in Toruń, Torun, Poland

Dear Professor Megha Kaviraj, Thank you for your support in reviewing our manuscript. We greatly appreciate your insightful comments. We are committed to incorporating your feedback to improve the manuscript, and we aim to meet your expectations with the revised version. Sincerely, Agnieszka Kalwasińska

 Answers for the Revewer's comments

 Answer 1: The Abstract has been changed accordingly

*Answer 2: The information regarding the soil used for the study along with its physicochemical properties has been added to the Materials and Methods section.*

*Answer 3: All data presented in the manuscript are in figures (Fig.1 – Fig.12), additionally, the details can also be inspected in Excel files in the repository RepOD https://doi.org/10.18150/10SRTX ( Kalwasińska, 2023).*

*Answer 4: The organism’s name was checked throughout the manuscript and all figures were improved accordingly.*

*Answer 5: The application of the carrier-based solid bacterial formulation with skimmed milk instead of a liquid formulation has been explained in the Materials and Methods section.*

*Answer 6: The future research has been adjusted accordingly.*

*Answer 7: The entire manuscript has been checked for spelling, and corrections were made.*
